# Development of a Mouse Model of Coccidioidomycosis Using an Inhalation Exposure System

**DOI:** 10.3390/jof11080599

**Published:** 2025-08-19

**Authors:** Jonathan Rodrigo Erlich, Priscila Rodriguez, Ka Pui Sharon Yau, Matthew Tate, Aaron F. Carlin, Joshua Fierer, Theo N. Kirkland, Hal M. Hoffman, Sinem Beyhan, Ben A. Croker

**Affiliations:** 1 Department of Pediatrics, University of California San Diego, La Jolla, CA 92093, USAp8rodriguez@ucsd.edu (P.R.); p1yau@health.ucsd.edu (K.P.S.Y.); hahoffman@health.ucsd.edu (H.M.H.); 2 Veterans Medical Research Foundation, San Diego, CA 92161, USA; mtate@ucsd.edu (M.T.); jfierer@health.ucsd.edu (J.F.); 3 Department of Medicine, University of California San Diego, La Jolla, CA 92093, USA; acarlin@health.ucsd.edu (A.F.C.); tkirkland@health.ucsd.edu (T.N.K.); 4 Department of Pathology, University of California San Diego, La Jolla, CA 92093, USA; 5 Department of Infectious Diseases, J. Craig Venter Institute, La Jolla, CA 92037, USA

**Keywords:** *Coccidioides*, aerosol exposure, inhalation, intranasal, coccidioidomycosis, fungal

## Abstract

*Coccidioides* species are thermally dimorphic fungal pathogens that cause coccidioidomycosis (Valley Fever) primarily in North and South America. *Coccidioides* grow as hyphae that differentiate into arthroconidia, which can be aerosolized upon soil disturbance, and inhaled by the mammalian host to cause pulmonary infections with occasional dissemination to other organs. In the context of mouse models, current methods of infection include intranasal, intravenous, and intraperitoneal delivery of the arthroconidia into mice. To explore an aerosol route of infection, we compared the intranasal method with aerosolization using the Glass-Col Inhalation Exposure System (IES). Infection with a dose of 2 × 10^6^ CFU/mL, nebulized in 5 mL of PBS, but not in water, was able to infect mice, albeit inconsistently, compared to intranasal challenge. Arthroconidia were detected inside the IES after the nebulization and decontamination cycles. These studies highlight some of the challenges with aerosolization of *Coccidioides* arthroconidia and serve as a reminder about biosafety considerations for use of the IES to aerosolize pathogens.

## 1. Introduction

*Coccidioides* are pathogenic dimorphic fungi endemic to the western United States, Central America, and South America, and a causative agent of coccidioidomycosis, a pulmonary disease commonly known as Valley Fever [1,2]. Infection occurs after inhalation of aerosolized arthroconidia released from soil disturbances such as weather events, construction, mining, and farming. While infections may be asymptomatic or produce mild self-limited flu-like symptoms, a subset of individuals develop pneumonia, and disseminated infections of peripheral organs and the central nervous system [3].

Rodents are naturally infected in endemic areas and laboratory mice are used commonly to model coccidioidomycosis. The intranasal (I.N.) route of infection is commonly employed for infecting laboratory mice with a wide range of pathogens including influenza, SARS-CoV-2, *Streptococcus pneumoniae*, *Histoplasma capsulatum*, and *Coccidioides* species [4,5,6,7,8,9,10]. This technique requires anesthesia of mice with ketamine/xylazine or isoflurane, followed by intranasal (I.N.) instillation of a pathogen suspended in phosphate-buffered saline (PBS).

*Coccidioides* is an airborne pathogen, and infection is initiated by the inhalation of dry aerosolized arthroconidia, not respiratory droplets. While I.N. infection models using arthroconidia suspended in PBS effectively induce infection in mice, they may not perfectly replicate the natural exposure route, and anesthetics can potentially influence the immune system and disrupt physiological homeostasis [11]. As an alternative method to I.N. infection models, inhalation exposure systems have been successfully used for some airborne pathogens, such as *Mycobacterium tuberculosis* and SARS-CoV-2 [12,13,14]. We hypothesized that such a system could be used to nebulize *Coccidioides* arthroconidia and would serve as a physiologically relevant infection model, while eliminating possible confounding effects of anesthesia. In addition, since the instrument can hold up to 50 mice, investigators could simultaneously infect multiple strains of mice that might respond differently to anesthetics without concern about the anesthesia being another variable.

In this study, utilizing the Glas-Col Inhalation Exposure System (IES), we tested if nebulization of arthroconidia would be a higher throughput and reproducible method to induce murine coccidioidomycosis. We compared IES nebulization to intranasal infection, evaluating disease-free survival, local and systemic infection burdens, and biosafety considerations. This study seeks to establish a refined roadmap for future *Coccidioides* infection models.

## 2. Methods

### 2.1. Mice

In this study, 8–20-week-old male and female C57BL/6J mice were housed in pathogen-free conditions with unrestricted access to food and water. Mouse experiments were conducted in an ABSL3 facility in accordance with institutional biosafety regulations and the regulatory standards of the Institutional Animal Care and Use Committee.

### 2.2. Fungal Cultures

The *Coccidioides immitis* RS fungal strain (BEI, NR-48942) was plated from frozen stocks and cultured on 2X Glucose Yeast Extract (2X GYE) agar plates (2% glucose [Sigma-Aldrich, Burlington, MA, USA, #G8270-5KG], 1% yeast extract [Gibco #DF210929] (Waltham, MA, USA), agar [Gibco #DF0145-17-0]). Cultures grew for 4–6 weeks at 30 °C to induce arthroconidium formation. Arthroconidia were harvested as previously described [15]. Harvested stocks were stored at 4 °C, and viability was periodically assessed by plating serial dilutions on 2X GYE agar, incubating for 4 days at 30 °C, and quantifying colony-forming units (CFUs).

### 2.3. Aerosolization of Pathogens

*Coccidioides immitis R.S.* was nebulized in 5 mL of either MilliQ H_2_O or PBS in the Glas-Col Inhalation Exposure System^TM^ (IES; Terre Haute, IN, USA)) under the following parameters: 25 min of nebulization, 40 min of cloud decay, and 15 min of decontamination. Mice or agar plates were placed in the chamber for the entire duration of the IES run after which plates were removed to a humidified incubator at 30 °C with a mixture of 5% CO_2_ and 95% ambient air. Mouse weights were monitored after exposure, and they were euthanized if and when they lost >15% of their starting weight for three days in a row, or more than 20% bodyweight, whichever came first.

### 2.4. Intranasal Infection Model

Mice were anesthetized (3–5% isoflurane at 5 L/min and O_2_ at 2 L/min) and infected with 100 colony-forming units (CFUs) intranasally in a total volume of 50 µL of PBS. Weight loss was monitored as above, and we used the same criteria for euthanasia.

### 2.5. Fungal Titers

Lungs and spleen were placed in 2 mL SafeLock microtubes (Eppendorf) containing 1 mL of PBS and a 5 mm stainless steel bead (Qiagen) for tissue disruption using a TissueLyser II (Qiagen). Samples were serially diluted and plated on 2× GYE agar plates that were incubated at 30 °C and colonies were enumerated.

### 2.6. Statistical Analysis

Data were represented as the geometric mean  ±  standard error of the mean (SEM). Comparison of survival curves were performed via Gehan–Breslow–Wilcoxon test. All statistical analyses were performed using GraphPad Prism 10.4.1 (San Diego, CA, USA). *p* < 0.05 was considered statistically significant.

## 3. Results

### Nebulization of Coccidioides Inconsistently Infected Mice

**Figure 1:**
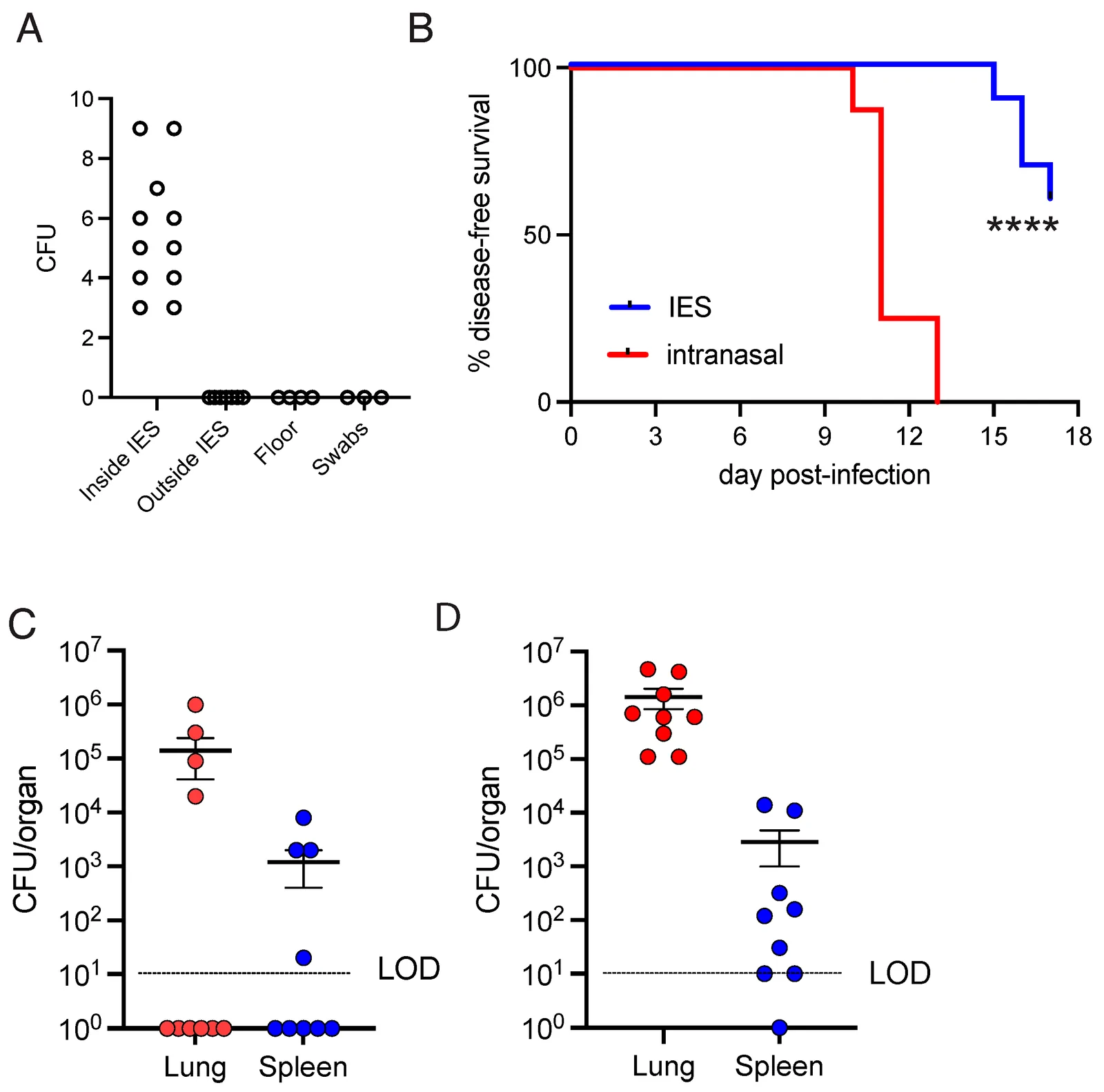
Aerosolization of *Coccidioides* arthroconidia to infect mice. (**A**) Open agar plates were placed as indicated throughout the chamber for the duration of the infection cycle, where *C. immitis* RS arthroconidia were nebulized in 5 mL of MilliQ H_2_O. Individual colonies counted from agar plates are represented. (**B**) Disease-free survival curves of mice infected by nebulizing 10^7^ CFUs in 5 mL of PBS or 100 CFUs intranasally. Mean  ±  SEM of *n*  =  8–10 mice are shown with statistical analyses performed via a Gehan–Breslow–Wilcoxon test. **** indicates *p* < 0.0001. (**C**,**D**) Lung or spleen homogenates of mice infected at (**C**) 1 × 10^6^ CFU/mL (5 mL total volume) of PBS collected 16 days P.I. or (**D**) 100 CFUs intranasally collected 15 days P.I. Data are represented as mean  ±  SEM of colony counts from *n*  =  8–10 spleens or lungs, where *n* represents one organ from one individual mouse.

To first confirm that *Coccidioides* could be nebulized into the aerosol chamber, *Coccidioides immitis* RS arthroconidia at 5000 CFU/mL were prepared in MilliQ H_2_O, which is the suggested diluent per manufacturer’s instructions, and then nebulized. Agar plates were placed inside baskets, outside of the baskets within the IES, outside of the IES, and on the floor adjacent to the IES to confirm containment of the pathogen within the IES, and then incubated at 30 °C. Only the plates from inside the IES had growth of the fungus; none of the plates that were outside of the IES or on the floor adjacent to the IES had any growth, demonstrating a low risk of leakage of the nebulized arthroconidia outside the IES (Figure 1A).

To examine the utility of the IES for infecting mice, we used C57BL/6J mice which are highly susceptible to coccidioidomycosis [16]. We compared our standard intranasal infection, to the IES starting with a nebulization dose of 5000 CFU/mL in 5 mL MilliQ H_2_O. We incrementally increased the number of aerosolized arthroconidia to 10^7^ CFU but no mice were infected when MilliQ H_2_O was used as the diluent (Figure 1 and Table 1).

Next, we tested PBS as a diluent since it is used in the I.N. route of infection. Surprisingly, when PBS was used as the diluent, after nebulization of 10^6^ CFU/mL, approximately 50% of mice were infected, and 80% were successfully infected at 2 × 10^6^ CFU/mL (Table 1). These data suggest that MilliQ H_2_O impairs infectivity by aerosolization but even in PBS a relatively high number of arthroconidia were required for efficient infection of mice.

To investigate the efficiency of the decontamination cycle of the IES after high dose (a total of 10^7^ CFU) of *Coccidioides* nebulization, the interior of the chamber, including baskets, trays, and walls, was swabbed for culture. Arthroconidia were detected from those surfaces, indicating that the decontamination cycle does not completely remove nebulized arthroconidia.

Next, we compared the two methods of infecting the lungs of C57BL/6J mice. Mice that were infected intranasally with 100 CFU reached criteria for euthanasia (>15% weight loss for >3 days or >20% weight loss) between days 10 and 13 after infection (Figure 1B). In contrast, mice infected by nebulizing 5 × 10^6^ CFU in PBS did not start to reach that endpoint until 15 days post-infection, and only 10 out of 19 mice were infected 17 days post-infection (Figure 1B). Using the same IES infection cycle, titers in the lung and spleen were assessed as a measure of the severity of the pneumonia and the amount of dissemination, respectively, at day 16 P.I, which correlated with endpoint disease as we defined (Figure 1C). From the IES cohort, only 4/9 IES mice had evidence of pulmonary infection, and all the infected mice had disseminated infections. In contrast, all mice that were infected intranasally with 100 CFU had evidence of infection in the lung and dissemination to the spleen (Figure 1D).

## 4. Discussion

Authentic modeling of human respiratory infections in mice remains an important goal for studying the pathogenesis of pulmonary infections. While intranasal infection provides an effective route for pulmonary infection, it is potentially complicated by the need to anesthetize mice, possible differences in lung distribution resulting from I.N. or aerosol infection, and potential for subtle changes to the immune response in the lung due to anesthesia. Consequently, in this study, we developed a protocol for nebulizing *Coccidioides*, a soil-derived and airborne fungal pathogen that is naturally acquired by inhaling arthroconidia. This study sought to establish a protocol for aerosolizing *Coccidioides* to better mimic the natural route of infection for humans.

Both intranasal and aerosol infection routes generated productive infections, but with the IES we could not infect any mice using the recommended fluid for suspending the inoculum, and only after switching to PBS and increasing the inoculum to 10^6^ CFU/mL did we infect about half of the mice that were exposed. Even that infection was less severe than the I.N. infection as measured by survival and by quantitative culture of the lungs and spleens. Disappointingly, there was still one log spread in the CFU of the IES-infected mice. PBS is not recommended by the maker of the instrument as the salts may crystallize in the nozzles, so this method of infecting mice with arthroconidia does not appear robust.

The lower colony counts of *C. immitis* from the lungs of mice infected by the aerosol route may have been due to a more vigorous innate immune response in their lungs, but more likely they were because a smaller number of organisms were inhaled than entered the lung after I.N. instillation. We are also concerned that despite exhaustion of the atmosphere in the IES device, there were residual viable *Coccidioides* on the inside of the IES post-decontamination cycle. Coccidioidomycosis is a biohazard for microbiologists in clinical laboratories [8], and the IES may need chemical disinfection after the end of a run so that subsequent users would not risk exposure to arthroconidia. There is no comparable risk when isoflurane or ketamine is used for I.N. infections inside a biosafety cabinet. Although it is an advantage to be able to infect many mice simultaneously using the IES, to ensure a high percentage will be infected, it is necessary to harvest >10^7^ arthroconidia, a formidable task.

These findings collectively highlight the complexity of modeling this respiratory fungal infection and the trade-offs between physiological relevance, experimental consistency, and safety. While the I.N. model offers uniform infection, it may exaggerate disease severity, potentially due to anesthesia-related immunomodulation, though this is a hypothetical risk. The IES has been used successfully to infect mice with other respiratory pathogens, but we were unable to use it to reliably infect mice by aerosolization. We were unable to find a protocol that was compatible with the instrument that uniformly infected the mice. In addition, the increased biohazards associated with residual contamination of the instrument, including the need to grow and harvest a large quantity of the infectious mold for each experiment and ensuring biosafety, is of significant concern. As it stands, we cannot recommend adoption of this model to study pulmonary coccidioidomycosis.

## Figures and Tables

**Table 1:** List of tests performed through nebulization of *Coccidioides immitis* RS arthroconidia.

Dose (CFU/mL)	Nebulized Dose (CFU)	Diluent	Test	Infected
5 × 10^3^	2.5 × 10^4^	H_2_O	Agar plates	Growth
5 × 10^3^	2.5 × 10^4^	H_2_O	Mice	1/49
5 × 10^4^	2.5 × 10^5^	H_2_O	Mice	0/50
2 × 10^6^	1 × 10^7^	H_2_O	Mice	0/50
2 × 10^6^	1 × 10^7^	PBS	Mice	4/5
1 × 10^6^	5 × 10^6^	PBS	Mice	10/19

## Data Availability

The original contributions presented in this study are included in the article. Further inquiries can be directed to the corresponding authors.
